# Diet-Induced Abdominal Obesity, Metabolic Changes, and Atherosclerosis in Hypercholesterolemic Minipigs

**DOI:** 10.1155/2018/6823193

**Published:** 2018-02-25

**Authors:** Ahmed Ludvigsen Al-Mashhadi, Christian Bo Poulsen, Karin von Wachenfeldt, Anna-Karin Robertson, Jacob Fog Bentzon, Lars Bo Nielsen, Jesper Thygesen, Lars Poulsen Tolbod, Jens Rolighed Larsen, Søren Kragh Moestrup, Björn Frendéus, Brynjulf Mortensen, Ludovic Drouet, Rozh H. Al-Mashhadi, Erling Falk

**Affiliations:** ^1^Department of Clinical Medicine, Aarhus University and Department of Cardiology, Aarhus University Hospital, Aarhus, Denmark; ^2^Truly Translational, Lund, Sweden; ^3^BioInvent International AB, Lund, Sweden; ^4^Department of Clinical Biochemistry, Rigshospitalet, University of Copenhagen, Copenhagen, Denmark; ^5^Department of Biomedical Engineering, Aarhus University Hospital, Aarhus, Denmark; ^6^Department of Nuclear Medicine and PET-Centre, Aarhus University Hospital, Aarhus, Denmark; ^7^Department of Cardiothoracic and Vascular Surgery, Aarhus University Hospital, Aarhus, Denmark; ^8^Department of Molecular Medicine, University of Southern Denmark, Odense, Denmark; ^9^Center for Diabetes Research, Gentofte Hospital, University of Copenhagen, Hellerup, Denmark; ^10^Institute of Vessels and Blood, Hospital Lariboisiere, Paris, France

## Abstract

**Background:**

Obesity and metabolic syndrome (MetS) are major risk factors for atherosclerotic diseases; however, a causal link remains elusive. Animal models resembling human MetS and its complications, while important, are scarce. We aimed at developing a porcine model of human MetS.

**Methods:**

Forty pigs with familial hypercholesterolemia were fed a high fat + fructose diet for 30 weeks. Metabolic assessments and subcutaneous fat biopsies were obtained at 18 and 30 weeks, and fat distribution was assessed by CT-scans. Postmortem, macrophage density, and phenotype in fat tissues were quantified along with atherosclerotic burden.

**Results:**

During the experiment, we observed a >4-fold in body weight, a significant but small increase in fasting glucose (4.1 mmol/L), insulin (3.1 mU/L), triglycerides (0.5 mmol/L), and HDL cholesterol (2.6 mmol/L). Subcutaneous fat correlated with insulin resistance, but intra-abdominal fat correlated *inversely* with insulin resistance and LDL cholesterol. More inflammatory macrophages were found in visceral versus subcutaneous fat, and inflammation decreased in subcutaneous fat over time.

**Conclusions:**

MetS based on human criteria was not achieved. Surprisingly, visceral fat seemed part of a healthier metabolic and inflammatory profile. These results differ from human findings, and further research is needed to understand the relationship between obesity and MetS in porcine models.

## 1. Introduction

Metabolic syndrome (MetS) is a cluster of insulin resistance (IR), impaired glucose tolerance, dyslipidemia (high triglycerides, low high-density lipoprotein cholesterol (HDL-C)), and hypertension often accompanying obesity. Especially, IR is considered a central part of MetS and type 2 diabetes, although the mechanisms are not fully understood [1]. Because MetS and type 2 diabetes in humans are associated with atherosclerotic cardiovascular disease, such as myocardial infarction, stroke, and peripheral artery disease, there is a constant interest in animal models of MetS and type 2 diabetes, with potential in translational research [2]. Such animal models must ideally combine atherosclerosis and type 2 diabetes, as these outcomes are ultimately of interest to understand and prevent in humans.

Recently, porcine models of MetS have gained attention. However, the task has proven demanding, as only select breeds develop more than trivial changes in MetS parameters and none develop frank type 2 diabetes in response to overfeeding and obesity [3–9]. This is depicted in Table 1 where a sample of current porcine models of MS is displayed. While some secondary detrimental effects of obesity have been observed, for example, nonalcoholic fatty liver disease [10, 11], it has not been demonstrated that obesity and metabolic changes in porcine models accelerate atherosclerosis. It should be noted that the studies in Table 1 differ on several key design parameters such as diet, duration, weight, and number of pigs and serve as an overview rather than strict comparison.

In humans, chronic low-grade inflammation involving the adipose tissue (AT) is believed to play a pivotal role in the development of MetS [12–14]. AT expansion is followed by macrophage infiltration, formation of crown-like structures, and increased inflammatory markers in plasma [15–20]. While obesity generally correlates with IR and type 2 diabetes, there is a stronger correlation between, specifically, visceral AT expansion and IR [1, 14, 21–23]. Whether porcine models of MetS display a similar pattern of inflammation and IR is currently unknown.

We investigated 40 minipigs known to develop human-like atherosclerosis spontaneously [24] and assessed the metabolic effects of a high fat + fructose diet (HFF) fed for 30 weeks. The pigs were part of a study investigating the effects of MLDL1278A, a human antibody against oxidized apolipoprotein B [25]. In this paper, we present the effects of obesity on the metabolic parameters used to define MetS in this porcine model. We also show the associations between obesity, atherosclerosis, and MetS parameters, and we report the inflammatory profile and distribution of ATs in these pigs. The differences and similarities to human pathology are relevant to consider in translational research in obesity and MetS.

## 2. Material and Methods

Forty atherosclerosis-susceptible familial hypercholesterolemia Bretoncelles Meishan (FBM) pigs were included in this study. FBM pigs have defective low-density lipoprotein (LDL) receptors and are prone to develop hyperlipidemia and atherosclerosis on high cholesterol diet [24]. In human females, menopause is an independent risk factor for MetS [26] and our pigs were all castrated females. The pigs were fed regular chow from birth until aged 6–11 months, after which they were fed ad libitum, 8 hours a day, for 7-8 months with a custom-made, calorie-dense HFF diet, consisting of 2% cholesterol, 22% fat (lard), and 18% fructose (Supplementary Figure 2). Following the development of severe obesity, the effect of a potential antiatherosclerotic drug (MLDL1278A) was tested in 20 pigs, while 20 pigs received placebo as previously reported [25]. Two pigs were excluded due to severe chronic lung disease discovered during autopsy, and in one this was also found in a computed tomography (CT) scan performed 12 weeks prior. Metabolic assessments were performed at three weeks before HFF diet, that is, W(−3), and at 18 weeks (W18) and 30 weeks (W30) on HFF diet as outlined in Table 2 and detailed in the online supplementary information (SI) (available here). The study was conducted in accordance with ILAR's “Guide for the Care and Use of Laboratory Animals,” and the Danish Animal Experiments Inspectorate approved the study.

### 2.1. Intravenous Glucose Tolerance Test (IVGTT)

Central and peripheral venous catheters were placed via the jugular vein and in the auricular vein, respectively, in 7 pigs at W(−3) before HFF diet and in all 40 pigs at time points W18 and W30 as previously described [25] (Table 2). After an overnight fast, with the catheters in place, the pigs were transferred to a containment cage. They settled for 20–30 minutes, and 3 baseline blood samples were collected to determine fasting insulin, glucose, and lipid levels. A glucose bolus (500 mg/kg) was infused through the peripheral venous catheter, and blood samples were drawn from the central venous catheter after 5, 10, 20, 30, and 60 minutes (SI).

We calculated the area under the curve (AUC) following glucose infusion and the homeostatic model assessment of insulin resistance (HOMA-IR) [27] from the fasting baseline values. We also computed the calculated insulin sensitivity index (CS_I_) [28]. This was done using a minimally modified formula:

(1) $$\begin{array}{c} {CS}_{I}=\frac{K_{G}}{\left(\Delta {AUC}_{INS}/\Delta T\right)}, \end{array}$$

where *K*_G_ is the glucose disposal rate (the slope of log[glucose]) from 5 to 60 minutes after glucose infusion, ΔAUC_INS_ is the AUC of insulin above baseline, and Δ*T* is equal to 55 minutes (modified from 40 in the original formula).

Due to technical difficulties, observations from 3 pigs (1 drug, 2 placebo) were excluded from the W18 samples. The 7 IVGTTs performed at time point W(−3), before HFF diet, were conducted at another facility, and the procedure differed in regard to handling and venous catheter placement (SI).

### 2.2. Assessment of Obesity and Adipose Tissue Distribution

Whole-body computed tomography (CT) scans were performed on all pigs 1–8 days prior to euthanization as described by Val-Laillet et al. [29] (see SI and Supplementary Figure 3). We measured intra-abdominal, subcutaneous AT, and total cross section. “Lean tissues” was obtained by subtracting total AT from the total cross section (Supplementary Figure 4). CT data files from one pig, in the placebo group, became corrupt and could not be analyzed.

### 2.3. Lipid Profiling

Fasting blood samples for assessment of total cholesterol, LDL cholesterol (LDL-C), HDL-C, and triglycerides were obtained at baseline before HFF diet W(−3) and after 18 and 30 weeks on HFF, W18 and W30. This was measured during the IVGTT procedure prior to glucose bolus (Table 2 and SI) [25].

### 2.4. Blood Pressure

Blood pressure was measured noninvasively before central venous catheter placement (time points T3 and T4) using an automated sphygmomanometer on the tail artery measuring heart rate and systolic, diastolic, and mean arterial pressure (SI).

### 2.5. Adipose Tissue and Inflammation

Subcutaneous AT was obtained at W18 and W30 by sample excision in the gluteal area of anesthetized pigs (for details see SI). In addition, at W30, we analyzed perivascular AT adjacent to the left anterior descending coronary artery (LAD), mesenteric AT in relation to the small intestine, and extraperitoneal AT. Perivascular AT sections were chosen based on the LAD site with the largest atherosclerotic plaque. Tissue sections were blinded for group (drug versus placebo). Macrophage phenotype was assessed by immunohistochemistry using two macrophage markers: CD163, a type B scavenger receptor and an anti-inflammatory macrophage-specific marker [30], and cathepsin S (CatS), a cysteine protease found in phagocytic cells and a proinflammatory serum marker associated with IR [31–33], inflammation, and atherosclerosis [34]. Five random high-power-field images were taken through a 20x objective using a camera-mounted microscope. The image files were then blinded for adipose tissue compartment, and positive cells were identified by applying a color threshold and counted in ImageJ (National Institutes of Health). Crown-like structures were defined as >50% of an adipocyte circumference occupied by CatS-positive cells or >3 CatS-positive cells encircling an adipocyte (SI). We visually searched through each image of the CatS slides and noted if there were any crown-like structure present [16, 35] (for details see SI).

### 2.6. Quantification of Atherosclerosis

The aorta, the right coronary artery, and the left iliac artery were stained *en face* with Sudan IV, and atherosclerosis burden was quantified as intimal surface lesion area in percentages of total vessel wall area [25, 36].

### 2.7. Statistical Analyses

Analyses were performed using GraphPad Prism® (GraphPad Software Inc., CA, USA) and STATA® (StataCorp LP, TX, USA). Between group comparisons were done using Student's *t*-test, Mann–Whitney test, or Wilcoxon matched-pairs signed rank test as appropriate. In one instance, multiple comparisons were tested using Kruskal-Wallis and corrected with Dunn's posttest (the inflammatory profile of the different AT). Correlations were computed using multivariable linear regression analyses, where the heteroscedasticity consistent covariant matrix was applied to obtain robust standard errors [37]. Before combining the two groups (drug versus placebo), slopes and intersects were tested for significance in traditional univariable regression. No differences were found. Statistical significance was accepted at *p* < 0.05, and trend was defined as *p* < 0.1.

## 3. Results

Because we found no effect of the drug on any of the parameters we investigated in this study, the drug and placebo groups were combined for further analyses, to increase statistical power. For an overview of the metabolic parameters, see Table 3. A comparison of three other porcine models of human MetS [3–5] is provided in Table 1.

### 3.1. Body Weight and Obesity

**Figure 1:**
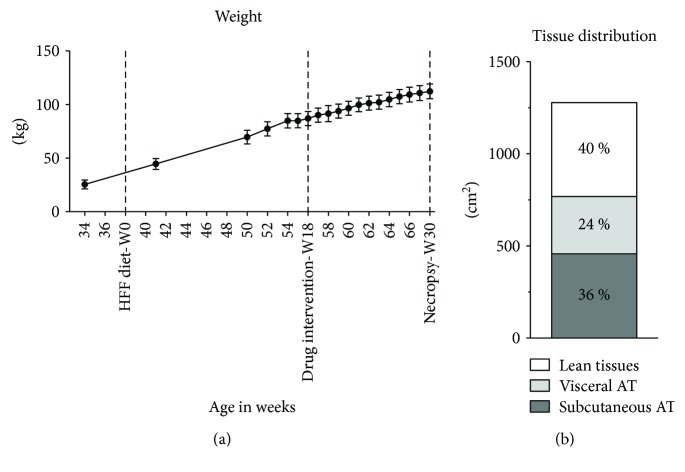
Body weight and fat distribution. (a) Weight gain measured throughout the study. (b) Distribution of abdominal fat depots as quantified by computed tomography scans at the level of the second lumbar vertebra. Bars represent 95% CI.

On regular chow, the pigs weighed 25.6 (±12.7, SD) kg on average and the weight increased to 112.3 (±20.8, SD) kg following 30 weeks on the diet (Table 3, Figure 1(a)). CT scans prior to euthanization showed that the high weight was predominately driven by fat gain as shown in Figure 1(b), where fat tissue represented 60% of the abdominal cross section, interquartile range (57%–64%).

During the final 12 weeks of the study (from W18 to W30), there was a statistically significant increase in body weight, fasting glucose and insulin, HOMA-IR, and AUCs for glucose and insulin (Table 3 and Supplementary Table 1). Similarly, CS_I_ decreased significantly by 22% (*p* = 0.001, Supplementary Table 1).

Three weeks prior to HFF diet, W(−3), we performed IVGTT on 7 pigs in a different facility from that in W18 and W30. Here, we found a discrepancy between the changes in fasting and IVGTT measurement over time, and that fasting glucose, but not HOMA-IR, decreased while CS_I_ also decreased (Supplementary Table 1).

### 3.2. Relationship of Obesity and Insulin Resistance

**Figure 2:**
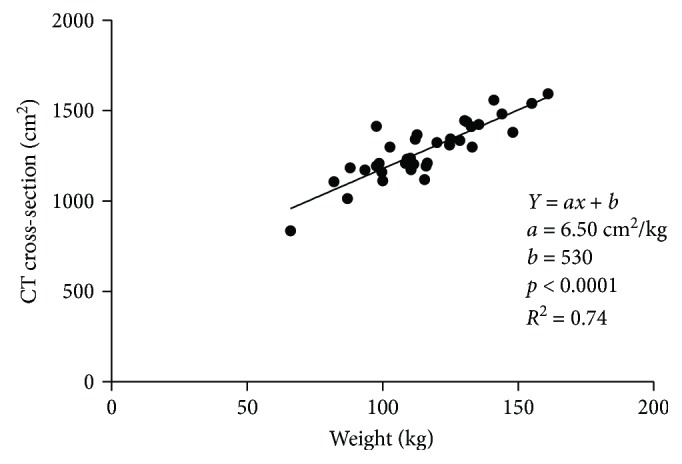
Weight correlated with total CT cross-sectional area.

By incorporating Val-Laillet's method of quantifying fat depots by CT scan, we were able to obtain reliable measures of obesity, evident through a strong correlation between weight and abdominal CT cross section (*R* ^2^ = 0.74, Figure 2). Similarly, in multivariable regression, weight was strongly, and independently, correlated to intra-abdominal AT, subcutaneous AT, and lean tissue (*R* ^2^ = 0.76, Table 4).

**Figure 3:**
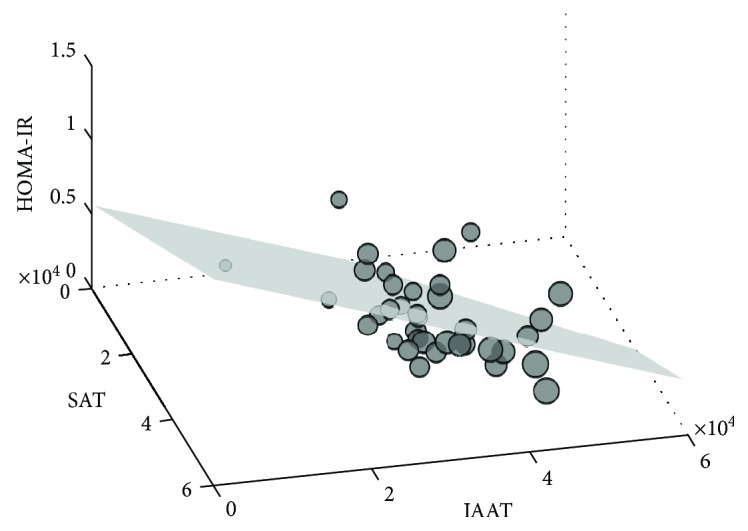
HOMA-IR correlated with subcutaneous and intra-abdominal AT. XYZ plot depicting the multivariable correlation for HOMA-IR. Size of markers represents weight.

The above parameters of obesity were correlated to IR in multivariable regression models. Surprisingly, we found that subcutaneous AT showed an independent *direct* correlation, while intra-abdominal AT showed an independent *inverse* correlation with HOMA-IR. Lean tissues did not correlate with HOMA-IR (*p* = 0.49, Table 4, Figure 3) nor did total body weight (*p* = 0.61). When fat depots were normalized to total CT cross section, the correlations were strengthened for subcutaneous AT (*p* = 0.01) as well as intra-abdominal AT (*p* = 0.038) with *R* ^2^ = 0.20.

Subcutaneous AT but not intra-abdominal AT (*p* = 0.20) correlated inversely to CS_I_ in a two-variable regression model. Adding lean tissues to the model did not improve the correlations, and weight did not significantly correlate with CS_I_ (*p* = 0.078). Excluding the two insignificant variables resulted in a univariable model of CS_I_ versus subcutaneous AT as shown in Table 4.

When fat depots were normalized to total CT cross section, the inverse correlation between subcutaneous AT and CS_I_ was strengthened (*p* = 0.010, *R* ^2^ = 0.19) but remained insignificant for intra-abdominal AT and CS_I_ (*p* = 0.58).

### 3.3. Lipid Profile

Compared to regular chow, HFF diet led to a substantial increase in total cholesterol, LDL-C, and HDL-C and a smaller increase in the LDL-C/HDL-C ratio and triglycerides (Table 3). Only small changes were seen during the final 12 weeks of the study. Lipid data is detailed in a previous publication [25].

Interestingly, we found that intra-abdominal AT, but not subcutaneous AT, correlated inversely with LDL cholesterol as well as total cholesterol (Table 4). No such correlation was found between obesity and triglycerides (*p* > 0.3).

### 3.4. Blood Pressure

In the 12 weeks between W18 and W30, the systolic blood pressure declined from 141 to 131 mmHg (*p* = 0.016; Table 3, Supplementary Table 1). Heart rate and diastolic and mean blood pressures did not change.

Subcutaneous AT was inversely correlated with mean arterial pressure (*p* = 0.043), and intra-abdominal AT trended towards a direct correlation with mean arterial pressure (*p* = 0.057).

### 3.5. Adipose Tissue and Inflammation

To quantify macrophages and to estimate the inflammatory profile of the ATs, we used CD163 and CatS as described above. We found neither a significant effect nor trend for an effect of MLDL1278A on the expression of CD163 or CatS in any of the AT compartments (data not shown).

**Figure 4:**
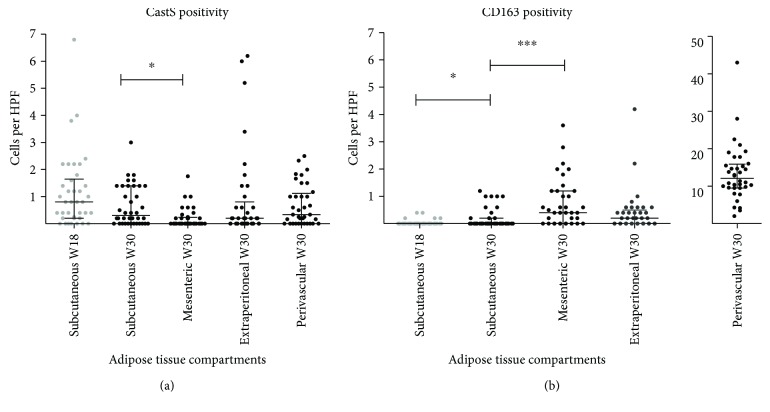
Macrophage polarization in adipose tissues. Number of cathepsin S-positive (a) and CD163-positive (b) cells per high-power field in subcutaneous AT (assessed at time point W18 and W30), mesenteric, extraperitoneal, and perivascular AT. CD163 in perivascular AT is displayed on a separate graph due to a difference by one order of magnitude. Significance was tested with Kruskal-Wallis and corrected with Dunn's posttest for multiple comparisons. ^∗^ *p* < 0.05; ^∗∗∗^ *p* < 0.001. Bars represent median ± interquartile range.

**Figure 5:**
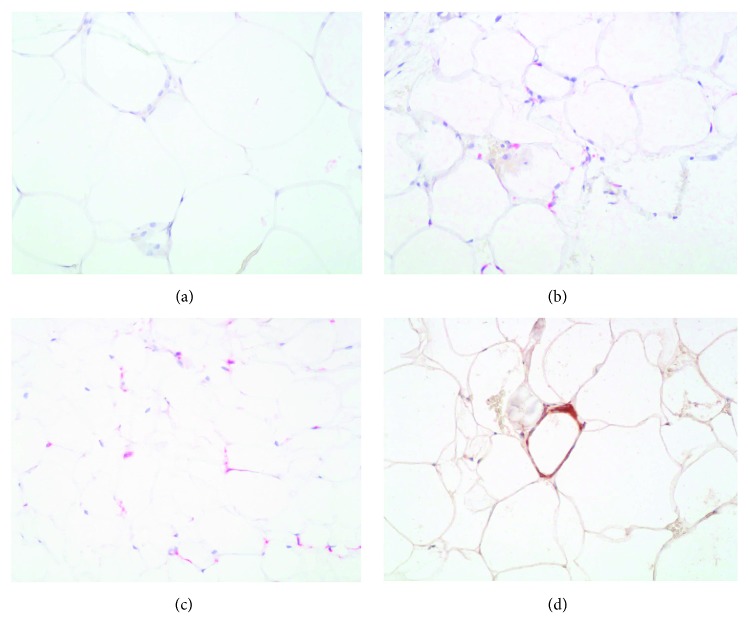
CD163- and CatS-positive cells in adipose tissues. (a–c) Subcutaneous (a, b) and perivascular (c) AT stained for CD163 (red) and counterstained with hematoxylin (blue). (a) Random high-power field with 0 positive cells. (b) Same section, after actively searching for an area with positive cells. (c) Perivascular AT with an average number of positive cells. (d) Crown-like structure of CatS-positive cells (brown).

During the final 12 weeks, CD163-positive cells increased in subcutaneous AT (*p* = 0.02) with a trend for reduction in CatS-positive cells (*p* = 0.07; see Figure 4(a)). This indicates a polarization of subcutaneous AT in an anti-inflammatory direction in response to ongoing obesity. Remarkably, at euthanization, mesenteric AT was characterized by a relatively low inflammatory cell count. We found a significantly lower density of CatS-positive cells and a significantly higher density of CD163-positive cells compared with subcutaneous AT (Figure 4(b)). Perivascular AT surrounding atherosclerotic lesions contained a substantially higher density of CD163-positive cells compared with the other adipose tissue compartments (>30-fold) (Figures 4(b) and 5(c)).

From the entire CatS data set comprising 38 pigs with 4 AT compartments per pig and 5 high-power fields per compartment, surmounting to approximately 760 high-power fields, we only found 19 crown-like structures in 18 high-power fields, an average of 1.45 crown-like structures/mm^2^.  Figure 5 shows slides of subcutaneous and perivascular AT with representative numbers of stained cells.

### 3.6. Relationship of Obesity, HOMA-IR, and CS_I_ to Atherosclerosis

We performed two sets of multivariable regression with atherosclerosis data. First, we correlated the disease burden, as quantified by en face, of each vessel bed (aorta, right coronary artery, and left iliac artery) against HOMA-IR, CS_I_, and cholesterol. In this setting, cholesterol but not HOMA-IR nor CS_I_ correlated with atherosclerosis burden. The correlation was significant between cholesterol and atherosclerosis in the aorta (*p* = 0.002; *R* ^2^ = 0.13) and trending for the iliac and right coronary arteries (*p* < 0.1). Next, we correlated the atherosclerosis burden of each vessel bed against intra-abdominal and subcutaneous AT. There were no independent correlations, consistently *p* > 0.3 (data not shown).

## 4. Discussion

After 30 weeks on nearly ad libitum HFF diet, the pigs became severely obese with >4-fold increased body weight and a predominant fat distribution of 60% (Figure 1(b)). We observed significant but small increases in fasting glucose, total cholesterol, LDL-C, HDL-C, and triglycerides. However, except for obesity, no parameters met the diagnostic criteria for human MetS, including IR and hypertension [1]. While the cutoff values of MetS parameters may very well differ between humans and pigs, or even between breeds, these physiological differences are important to explore further. In contrast to humans, intra-abdominal AT was not directly correlated with any MetS parameter. In fact, it was inversely correlated with IR (HOMA-IR) and cholesterol. Subcutaneous AT, on the other hand, was directly correlated to IR and cholesterol. In agreement with this, we found that macrophage density and phenotype in adipose tissues differed from that described in human obesity and that IR did not correlate with atherosclerosis. It has been demonstrated that porcine models express variable metabolic changes in response to high calorie intake and development of obesity and may even be categorized in metabolically healthy versus unhealthy obesity [8, 9]. The changes in MetS parameters of our obese FBM pigs are similar to those found in other porcine models of metabolically “unhealthy” obesity (Table 1) [8, 9]. However, the phenotypes and correlations of obesity, inflammation, and IR found in the FBM pigs are different from human findings. This variable decoupling of obesity and MetS could very well provide us with clues to the human pathology and deserves further attention.

### 4.1. No Confounding Drug Effects

The experimental setup described in this paper was designed as a substudy to one testing the effects of a human antibody against oxidized apolipoprotein B on atherosclerosis [25]. The drug (MLDL1278A) has previously been shown to inhibit the development of atherosclerosis and promote stabilization of atherosclerotic plaques in mice [38, 39]. Furthermore, the drug apparently also improved insulin sensitivity in obese Rhesus macaques [40]. However, it failed to affect atherosclerosis plaque burden [25] and to meet the primary endpoint in a phase IIa clinical trial [41]. In the current substudy (obese pigs as model for human MetS), we found no effects of the drug on any of the components of MetS, which allowed us to combine the treatment and placebo groups in order to increase statistical power.

### 4.2. Increased Insulin Resistance but No Glucose Intolerance

To induce IR, a substantial amount of fructose was added to the diet [42, 43] and the pigs were fed ad libitum in daytime hours to promote obesity. Consequently, the FBM pigs became severely obese with an average weight of 112.3 kg at euthanization. AT accounted for ~60% of the total abdominal area, indicating severe abdominal obesity. In comparison, FBM pigs (castrated males) included in a previous study had a mean body weight of ~43 kg after being on a semirestricted high-fat diet for 18 weeks [24]. At the same age, our pigs weighed an average of 91.6 kg on HFF diet.

We used several methods to assess glucose intolerance and IR, including fasting insulin and glucose (HOMA-IR), IVGTT (AUC analyses), and CS_I_. HOMA-IR increased by 24% over the last 12 weeks, reaching 0.59. In comparison, in a general healthy nondiabetic human population, HOMA-IR ranged from 0.63 to 4.14 (5th to 95th percentiles) [44], that is, the mean HOMA-IR for our severely obese pigs remained below the 5th percentile of nondiabetic human subjects.

We performed IVGTT on the pigs at different time points. However, the baseline W(−3) measurements in a small subgroup of pigs (*n* = 7) are not directly comparable to those at W18 and W30 due to different anesthetic and handling procedures. These differences may explain the small decrease in fasting glucose (12%) and concomitantly decrease (78%) in insulin sensitivity as measured by CS_I_ (Supplementary Table 1).

During the final 12 weeks of the study, body weight increased substantially together with increasing IR (HOMA-IR, AUC_Insulin_) and decreasing insulin sensitivity (CS_I_). However, the changes were modest, if not trivial. While AUC_glucose_ increased, plasma glucose still returned to baseline values 120 minutes after glucose loading (data not shown).

The interpretation of the abovementioned changes is complicated by the inherent pitfall of using IVGTT-based AUC calculations to quantify IR and glucose intolerance. In this setting, weight-adjusted glucose infusion leads to overdosage of obese pigs when compared to lean pigs of similar weight due to lower water content in fat tissue versus other tissues. Consequently, more obese pigs will have higher AUCs for glucose and thereby also insulin. While AUC measurements are less than ideal, they are the only estimation of peripheral IR used in other porcine models. We overcame the shortcomings of standard AUC calculations by using the CS_I_, which represents glucose uptake velocity per concentration of insulin above basal value. The CS_I_ value is independent of the absolute concentrations of glucose and insulin and reflects the “efficiency” of insulin in promoting glucose uptake. The CS_I_ model has been validated in human subjects and shown to correlate strongly with the gold standard euglycemic clamp test (*R* ^2^ = 0.82) [28]. While the application of CS_I_ calculation adds credibility to our findings, it demonstrates how modest the true change in IR is in response to severe obesity.

Interestingly, feeding standard low-fat minipig chow to Göttingen minipigs ad libitum (versus restrictively) led to obesity with lower fasting plasma glucose and insulin and higher insulin sensitivity [45].

### 4.3. Consistent Correlations between Insulin Resistance and Fat Distribution Contrasting Human Findings

It is apparent that not all obese humans develop MetS or cardiovascular disease. Obesity is a heterogeneous condition, and this is underlined by the weak correlations between insulin resistance and measures of obesity (weight, BMI, fat depot sizes, etc.) [23]. We did not expect all or most of our pigs to develop diabetes or even MetS, but it seems reasonable to expect that the direction of the correlations should be similar, if the underlying pathologies were to be similar. However, our findings were not in concordance with human studies.

In humans, visceral rather than subcutaneous AT is considered to play the major role in the development of obesity-related IR [1, 14, 21–23]. We found the opposite correlations in FBM pigs where intra-abdominal AT was *inversely* correlated to hepatic IR (HOMA-IR) and subcutaneous AT was *directly* correlated to both hepatic and whole body IR (i.e., inversely to CS_I_). These correlations were strengthened, when relative values were used, that is, fat depots as percentages of entire CT cross sections.

While the correlations were weak, they were in the same order of magnitude as similar analyses performed in human studies [23], and they may explain why FBM pigs are so resistant to obesity-related IR; in that, visceral AT may not impose the same negative consequences in FBM pigs as in humans.

We are unaware of similar correlation analyses conducted in other porcine models of MetS.

### 4.4. Unremarkable Dyslipidemia, Mimicking Other Porcine Models

In humans, plasma concentrations of triglycerides and HDL-C correlate inversely [46], and the “atherogenic” dyslipidemia commonly associated with MetS is characterized by raised triglycerides (>1.7 mmol/L) and low HDL-C (<0.9–1.29 mmol/L). In the FBM pigs, triglycerides did increase with obesity but remained low (mean ≤ 0.5 mmol/L), and HDL-C increased substantially from 0.7 mmol/L to 2.6 mmol/L. High HDL-C is to be expected in pigs on a cholesterol-rich diet, but the low triglyceride levels are remarkable considering the high amount of fructose added to the diet with nearly ad libitum feeding, and the successful development of severe abdominal obesity, which, in turn, did not correlate with triglycerides levels.

Interestingly, intra-abdominal AT seemed to correlate inversely with LDL-C as well as total cholesterol in our FBM pigs. While we cannot infer causality, this indicates that intra-abdominal fat deposition in pigs is not part of a detrimental metabolic phenotype of obesity.

In a recent study, diet-induced obesity in Ossabaw and Göttingen minipigs was not associated with hypertriglyceridemia but only led to a nonsignificant increase in triglycerides from 0.3 to 0.5 mmol/L [47]. The resistance of pigs to diet- and obesity-induced hypertriglyceridemia is not well understood but could be related to the lack of functionally effective cholesteryl ester transfer protein (CETP) [48], extrahepatic lipogenesis [49], or any of the other ways; lipoproteins and their metabolism differ in pigs and man [48, 50].

### 4.5. No Sign of Hypertension

With a final blood pressure of 131/66 mmHg, our severely obese pigs did not become hypertensive according to human reference range. In fact, the blood pressure tended to decline during the final 12 weeks of the study. Although the reliability of tail cuff measurements on anesthetized pigs may be questioned, it seems reasonable to conclude that the blood pressure did not increase during the final 12 weeks of the study despite a substantial increase in body weight. Remarkably, mean blood pressure was inversely correlated to subcutaneous AT.

### 4.6. Surprising Inflammatory Phenotype in Adipose Tissues

In this study, we found several key differences in the inflammatory profile of ATs from obese FBM pigs and those reported from human studies.

Firstly, we found a higher density of CatS-positive and a lower density of CD163-positive cells in subcutaneous versus mesenteric AT (a component of visceral AT). That is, there were more proinflammatory and fewer anti-inflammatory cells in subcutaneous compared to visceral AT.

This stands in contrast to human observations in which visceral AT is considered more proinflammatory and critical in the development of MetS [1, 14, 21].

Secondly, by repeating subcutaneous AT biopsies at W18 and W30, we found a significant increase in *anti*-inflammatory cells and a trend towards decrease in proinflammatory cells during the final 12 weeks on obesogenic diet. This indicates a stagnant inflammatory phenotype despite ongoing weight gain and progressive obesity.

Thirdly, we found very few CatS-positive crown-like structures. Only 2.4% of our high-power fields had 1 or more crown-like structures. In contrast, Bigornia et al. [51] found CD68-positive crown-like structures in 67% of their high-power fields from human fat using a similar high-power field size to ours (20x objective on a light microscope). This is concordant with differences in the inflammatory activity of ATs between man and pig.

It appears that the intra-abdominal AT is characterized by an *anti-*inflammatory profile relative to the subcutaneous AT. This is consistent with, and may even be causally linked to the surprising correlations between obesity and MetS parameters, most importantly IR. Thus, intra-abdominal AT expansion in the pigs is seemingly part of a beneficial phenotype of obesity.

A possible explanation could be found in many porcine strains inherent propensity towards obesity. Millenniums of selection pressure may have lead to a healthier, more viable phenotype of obesity with less inflammation and little metabolic disturbance.

### 4.7. No Correlation between Insulin Resistance and Atherosclerosis

Considering that MetS is a major risk factor for atherosclerotic vascular disease in humans [2], we expected that IR would accelerate atherogenesis and thus correlate positively with the final amount, or burden, of atherosclerosis. We could however not document such an effect independent of cholesterol. These findings further question the assumed link between inflammation, MetS, and atherosclerosis in the FBM pig and call for similar analyses to be conducted in other porcine MetS models for a better understanding.

### 4.8. Strengths and Limitations

Major strengths of the study are the large sample size and long-term nearly ad libitum feeding with a fat-, fructose-, and energy-rich diet without confounding cholate, leading to severe abdominal obesity. The unusually large sample size and feeding duration led to good statistical power, which allowed the detection of even small changes and weak correlations. Due to the logistical circumstances of the study, a lean control group of pigs on regular diet was not included, and therefore we cannot conclude on causality links between feeding and metabolic changes with certainty. However, a control group is not necessary to conclude that abdominal obesity in FBM pigs is not associated with metabolic dysfunction. Furthermore, the final correlation analyses were cross-sectional in nature, covering a wide range of body weights and obesity.

## 5. Conclusion

Based on the cut-points used to define MetS in humans, severely obese FBM pigs did not meet the diagnostic criteria for MetS despite a >4-fold weight gain. This is similar to previous findings in other obese pigs. The explanation may be found in our data. The FBM pig expresses a different phenotype of obesity with regard to inflammation, IR, and atherosclerosis compared to man. We found that intra-abdominal AT had a lower content of *proinflammatory* cells and a higher content of *anti*-inflammatory compared to subcutaneous AT. And intra-abdominal AT was correlated with a healthier metabolic phenotype, that is, reduced IR and cholesterol and insulin resistance did not correlate with atherosclerosis. While porcine models of MetS would be of great benefit, these differences may explain the difficulties experienced so far and a better understanding of porcine pathophysiology is needed. We encourage future studies to include markers of inflammation, fat distribution, and the use of the calculated insulin sensitivity index (CS_I_) in the assessment of porcine MetS. Our data shows fundamental differences in the relationship between obesity, inflammation, metabolic disturbances, and atherosclerosis in FBM pigs compared to man. A better understanding of these factors in other porcine models may provide valuable insights on the human metabolic syndrome.

## Figures and Tables

**Table 1:** Current competing porcine models of metabolic syndrome.

	Ossabaw pig 3		Göttingen pig 5		Iberian pig^b^ 4	
	Obese group		Obese group		Obese group	

Obesogenic diet	2.0% cholesterol, 17% hydrogenated soybean oil, 2.3% corn oil, 0.7% sodium cholate		32% crude fat, 17% protein		Ad libitum: 3.7% saturated fat, 2.8% polyunsaturated fat, 15.1% crude protein	

Control diet	Lean group		Lean group		Lean group	
	Standard chow		Standard chow		Standard chow	
	Lean (*n* = 9)	Obese (*n* = 8)	Lean (*n* = 6)	Obese (*n* = 6)	Lean (*n* = 4)	Obese (*n* = 6)

Duration	9 weeks		5 weeks		100 days	
Weight (kg)	29	45^a^^∗^	21	27^a^^∗^	209	251^a^^∗^
Fasting glucose (mmol/L)	<5.5	<5.5	4.4	4.5	<4.4	<4.4
Fasting insulin (mU/L)	<20	<20	11	14	<1	<1
HOMA-IR	—	—	2.1	2.9	<0.3	<0.3
Insulin AUC^c^ (mU/L × min)	2579	3811^∗^	1923	2895^∗^	—	—
Glucose AUC^c^ (mmol/L × min)	NS	NS	578	636	—	—
Systolic blood pressure (mmHg)	113	137	—	—	150	170^a^
Diastolic blood pressure (mmHg)	70	92	—	—	95	115^∗^
Mean blood pressure (mmHg)	79	107^∗^	—	—	112	137^∗^
Triglycerides (mmol/L)	0.42	0.57^∗^	0.13	0.24^∗^	0.24	0.32^∗^
Cholesterol						
Total (mmol/L)	4.1	12.4^∗^	1.8	2.0	1.7	2.9^∗^
HDL (mmol/L)	1.2	1.0^a^	0.8	1.0^∗^^a^	0.8	0.72^a^
LDL (mmol/L)	2.7	11^∗^	—	—	1.2	1.6^∗^
LDL/HDL ratio	2.5	11^∗^	—	—	1.5	2.3^∗^

NS: data not provided but reported as nonsignificant difference. ^a^Changes fulfill human criteria of metabolic syndrome. ^b^All data points read from published figures. ^c^IVGTT in all but Iberian pig (oral GTT). 60 minutes AUC in Ossabaw pigs and 120 min AUC in Göttingen and Iberian pigs 2 g/kg orally; ^∗^ *p* < 0.05.

**Table 2:** Study timeline. Varying metabolic assessments performed at time points W(−3) to W(30). At W(−3), we performed IVGTT on *n* = 7 pigs. At W18, we performed IVGTT and subcutaneous fat biopsies on all pigs. At W30, we performed IVGTT, extra- and intra-abdominal fat biopsies, and full-body computed tomography scans on all pigs.

			W(−3)	W0	W18	W30
Age in weeks	0	29	35	38	56	68
Diet	Chow			HFFD		
Intervention		Castration			Drug trial (MLDL1278A)	CT-scans
			Fasting lipids (*N* = 38)		Fasting lipids (*N* = 38)	Fasting lipids (*N* = 38)
			IVGTT (*N* = 7)		IVGTT (*N* = 37)	IVGTT (*N* = 38)
					Blood pressures	Blood pressures
					Subcutaneous AT biopsies	Subcutaneous AT biopsies
						Necropsy

**Table 3:** Characteristics of metabolic parameters in the FBM pigs.

	FBM pig		

Obesogenic diet	Ad libitum: 2.0% cholesterol, 22% fat (lard), 18% fructose		
Control diet	No control diet		
	W(−3)	W18	W30
	(*n* = 38)		

Duration	33 weeks		
		12 weeks	
Weight (kg)	25.6	87	112^∗^
Fasting glucose (mmol/L)		3.9 (3.7–4.2)	4.1^∗^ (3.9–4.3)
Fasting insulin (mU/L)		2.6 (2.2–3.2)	3.1^∗^ (2.5–4.4)
HOMA-IR		0.46 (0.36–0.58)	0.57^∗^ (0.43–0.86)
Insulin AUC (mU/L × min)		862 (671–1048)	1189^∗^ (915–1463)
Glucose AUC (mmol/L × min)		862 (±15)	948 (±17)^∗^
Systolic blood pressure (mmHg)		141 (127–150)	131^∗^ (113–141)
Diastolic blood pressure (mmHg)		68 (±13)	66 (±14)
Mean blood pressure (mmHg)		93 (86–101)	91 (73–97)
Triglycerides (mmol/L)	0.4 (0.4–0.5)		0.5^∗^ (0.4–0.8)
Cholesterol			
Total (mmol/L)	4.1 (±0.3)		16 (±1.1)^∗^
HDL (mmol/L)	0.7 (±0.1)		2.6 (±0.3)^∗^
LDL (mmol/L)	3.1 (2.4–3.8)		14^∗^ (11–15)
LDL/HDL ratio	3.9 (2.8–5.7)		5.4^∗^ (3.6–7.5)

For detailed statistical information, see supplementary information. Data is presented as mean (±SD) or median (interquartile range) where appropriate; ^∗^ *p* < 0.05.

**Table 4:** Adipose tissues and metabolic correlations.

Model	Coefficients	Unit	Robust SE	*p* value	*R* ^2^
Weight	= *a* ∗ subcutaneous AT + *b* ∗ intra-abdominal AT + *c* ∗ lean tissues + *k*				
	*a*	0.15 kg/cm^2^	0.032	**0.008**	**0.76**
	*b*	0.076 kg/cm^2^	0.027	**0.000**	
	c	0.10 kg/cm^2^	0.026	**0.000**	
	*k*	−30.9 kg	16.3	**0.067**	

CS_I_	= *a* ∗ subcutaneous AT + *k*				
	*a*	−3.10*E* − 05 au	1.36*E* − 5	**0.029**	**0.15**
	*k*	0.031 au	0.0065	**0.000**	

HOMA-IR	= *a* ∗ subcutaneous AT + *b* ∗ intra-abdominal AT + *k*				
	*a*	0.0014 au	0.00052	**0.013**	**0.11**
	*b*	−0.0017 au	0.00081	**0.040**	
	*k*	0.55 au	0.22	**0.018**	

LDL-C	= *a* ∗ subcutaneous AT + *b* ∗ intra-abdominal AT + *k*				
	*a*	0.0021 mmol/(L·cm^2^)	0.0054	0.693	0.16
	*b*	−0.019 mmol/(L·cm^2^)	0.0077	**0.019**	
	*k*	18.2 mmol/L	1.98	0.000	

Total cholesterol	= *a* ∗ subcutaneous AT + *b* ∗ intra-abdominal AT + *k*				
	*a*	0.0026 mmol/(L·cm^2^)	0.0058	0.658	0.18
	*b*	−0.024 mmol/(L·cm^2^)	0.0091	**0.013**	
	*k*	22.58 mmol/L	2.65	0.000	

Mean arterial pressure	= *a* ∗ subcutaneous AT + *b* ∗ intra-abdominal AT + *k*				
	*a*	−0.077 mmHg/cm^2^	0.036	**0.043**	0.12
	*b*	0.10 mmHg/cm^2^	0.052	0.057	
	*k*	89.0 mmHg/cm^2^	16.4	0.000	

## Abbreviations

AT: Adipose tissue
CatS: Cathepsin S
CS_I:_: Calculated insulin sensitivity index
CT: Computed tomography
FBM: Familial hypercholesterolemia Bretoncelles Meishan
HFF diet: High fat + fructose diet
HOMA-IR: Homeostatic model assessment of insulin resistance
IR: Insulin resistance
LAD: Left anterior descending coronary artery
MetS: Metabolic syndrome
SI: Supplementary information
W(−3): Three weeks prior to high fat + fructose diet
W18: 18 weeks following high fat + fructose diet
W30: 30 weeks following high fat + fructose diet.

## Supplementary Materials

Supplementary Materials Supplementary Table 1: supplementary statistical results: effects of drug and time/age. Supplementary Figure 1: study timeline—interventions and assessments. Supplementary Figure 2: high fat + fructose diet (HFFD). Supplementary Figure 3: identifying the P2 site by computed tomography imaging. Top: 3D reconstruction of the FBM-pig spinal column and ribcage. The red dot marks the P2 site corresponding to vertebra L2 right below the last true rib. Bottom: typical cross section at the L2 level of an obese FBM pig. Supplementary Figure 4: measurement of adipose tissue compartments. Left column: cross-sectional computed tomography images at the vertebra L2 level. Right column: thresholding binary images depicting AT (black). Top row: total AT. Subcutaneous AT is marked as a ROI in order to obtain the mean and SD values for AT in the pig. Middle row: intraperitoneal AT (visceral AT). Bottom row: intra-abdominal AT.
