# Supplementary material for: Diet-Induced Abdominal Obesity, Metabolic Changes, and Atherosclerosis in Hypercholesterolemic Minipigs
Source: J Diabetes Res. 2018 Feb 25;2018:6823193. doi: 10.1155/2018/6823193 (PMC5845503; doi:10.1155/2018/6823193)
Supplement: Supplementary Materials — Supplementary Table 1: supplementary statistical results: effects of drug and time/age. Supplementary Figure 1: study timeline—interventions and assessments. Supplementary Figure 2: high fat + fructose diet (HFFD). Supplementary Figure 3: identifying the P2 site by computed tomography imaging. Top: 3D reconstruction of the FBM-pig spinal column and ribcage. The red dot marks the P2 site corresponding to vertebra L2 right below the last true rib. Bottom: typical cross section at the L2 level of an obese FBM pig. Supplementary Figure 4: measurement of adipose tissue compartments. Left column: cross-sectional computed tomography images at the vertebra L2 level. Right column: thresholding binary images depicting AT (black). Top row: total AT. Subcutaneous AT is marked as a ROI in order to obtain the mean and SD values for AT in the pig. Middle row: intraperitoneal AT (visceral AT). Bottom row: intra-abdominal AT. [file 6823193.f1.pdf]

Supplementary Information

Diet-induced abdominal obesity, metabolic changes and atherosclerosis in hypercholesterolemic minipigs

Ahmed Ludvigsen Al-Mashhadi,[1]* Christian Bo Poulsen,[1]* Karin von Wachenfeldt,[2] Anna-Karin Robertson,[3] Jacob Fog Bentzon,[1] Lars Bo Nielsen,[4] Jesper Thygesen,[5] Lars Poulsen Tolbod,[6] Jens Rolighed Larsen,[7] Søren Kragh Moestrup,[8] Björn Frendéus,[3] Brynjulf Mortensen,[9] Ludovic Drouet,[10] Rozh Husain Al-Mashhadi,[1] Erling Falk[1]

[1]Department of Cardiology, Aarhus University Hospital, and Institute of Clinical Medicine, Aarhus University, Aarhus, Denmark; [2]Truly Translational, Lund, Sweden; [3]BioInvent International AB, Lund, Sweden; [4]Department of Clinical Biochemistry, Rigshospitalet, Copenhagen, Denmark; [5]Department of Biomedical Engineering, Aarhus University Hospital, Aarhus, Denmark; [6]Department of Nuclear Medicine and PET-Centre, Aarhus University Hospital, Aarhus, Denmark; [7]Department of Cardiothoracic and Vascular Surgery, Aarhus University Hospital, Aarhus, Denmark; [8]Department of Molecular Medicine, University of Southern Denmark, Odense, Denmark; [9]Center for Diabetes Research, Gentofte Hospital, University of Copenhagen, Hellerup, Denmark; [10]Institute of Vessels and Blood, Hospital Lariboisiere, Paris, France.

* Both authors contributed equally.

Corresponding author:
Ahmed L. Al-Mashhadi
Norrebrogade 44
DK-8000 Aarhus C
Denmark
Phone: +45 6165 6277
E-mail: Al-Mashhadi@dadlnet.dk

Abbreviations

AT	Adipose tissue
CatS	Cathepsin S
CS_I	Calculated insulin sensitivity index
CT	Computed tomography
CVC	Central venous catheter
FBM	Familial hypercholesterolemia Bretoncelles Meishan
HOMA-IR	Homeostatic Model Assessment of Insulin Resistance
HFFD	High fat+fructose diet
HPF	High power field
IHC	Immunohistochemistry
IR	Insulin resistance
MetS	Metabolic syndrome
PVC	Peripheral venous catheter
W(-3) – W30	three weeks before – 30 weeks after high fat +fructose diet

Supplementary Methods

Central venous catheter (CVC) placement

Central (CVC) and peripheral venous catheter (PVC) were placed as previously described[1] at weeks W18 and W30 in our research facility in Denmark.

At the W(-3) time point, we selected 7 of the oldest pigs (44-46 weeks of age) for a baseline IVGTT at a research facility in France. These pigs fasted overnight, and were then sedated with intra-muscular ketamine (10 mg/kg) and facemask with isoflurane (1%) for CVC placement. The pigs awoke from sedation shortly hereafter, and IVGTT was performed 2-3 hours after CVC placement. The procedure at W(-3) did not differ from those at W18 and W30 in other aspects.

Intravenous glucose tolerance test

15-45 hours following the CVC and PVC placement and after 17-20 hours of fasting, we lured the pigs from their pens into a containment cage. The adhesive bandaging was cut open to expose the extension cord of the CVC. An extension cord was connected to the PVC and flushed through with heparinized isotonic NaCl saline. All blood samples were collected from the CVC after which the CVC was flushed with 3mL of heparinized NaCl. Prior to each blood sample 4-5 mL of blood was drawn and discarded to prevent dilution of the actual samples. 3 samples were collected for baseline assessment at minutes -10, -5, and -0. A glucose bolus of 500mg/kg via a glucose solution of 200mg/mL = 2.5mL/kg was then infused using the PVC in 1-3 minutes and subsequently flushed with 10 mL of isotonic NaCl saline. 6 samples were collected following the glucose injection at minutes 5, 10, 20, 30 and 60. For each sample time point, 2mL was added to a NaF-KOx tube *(BD Vacutainer® ref 368920)* and 2mL was added to an EDTA tube *(BD Hemogard ™ Vacutainer ® ref 367841).* Samples were inversion mixed and kept on ice water and centrifuged (10m at 2000xG) within 30 minutes of collection. Samples were then transferred to Eppendorf ® tubes and stored in -80C freezer. At each time point venous glucose values were measured using a glucometer (Accu-check® Aviva Cat/TYP 05911974002). The test strips *(Accu-check® Aviva ref 05987431170)* used for the entirety of the study had lot number (490330). For the W30 measurements, all values were triple tested, and the average of these values were used in analyses. The triple testing was done with 3 different lot numbers, one of which was the original *(490330).*

For data analyses we used the Accu-check® measurements of plasma glucose, while the plasma samples (EDTA tube) were used to measure insulin levels *(Bioinvent, Lund, Sweden, using Mercodia® Insulin ELISA kit)*

Blood pressure

We measured blood pressures (BP) non-invasively in conjunction with CVC placement (W18 and W30). After the pigs were anesthetized and positioned on the surgical bed in the supine position, a neonatal sized cuff connected to an automated sphygmomanometer (GE Healthcare: Carescape® V100 Vital Signs Monitor) was placed around the proximal part of the pigtail, thus measuring the systolic and diastolic blood pressure and heart rate from the tail artery. This was done as early in the procedure as possible, usually within minutes after positioning. It was strived to obtain at least 3 measurements from each pig. This was occasionally not achieved, most likely due to the small tail circumferences of the pigs. Mean arterial pressure (MAP) was derived as MAP = (2 x diastolic pressure + systolic pressure)/3.

Adipose tissue and inflammation

We analyzed the perivascular adipose tissue (AT) adjacent to the left anterior descending coronary artery. The procedure for tissue extraction and ex-vivo handling is previously described[1]

We also analyzed abdominal subcutaneous AT, mesenteric AT in relation to the small intestine and extra-peritoneal AT. The subcutaneous AT biopsies were taken in conjunction with the CVC placements (W18 and W30). At week 18 on the diet (W18) a 3 cm long <0.5 cm thick boat shaped sample was excised from the *right* lower back of the animal, and at W30 from the *left* lower back of the animal. The samples were immersion-fixed in formalin for 9-18 hours and then stored in cold PBS. All the formalin-fixated samples were paraffin-embedded. We performed IHC analyses on all these samples using two markers: CD163 and Cathepsin S (CatS). Mesenteric AT and extraperitoneal AT were taken in conjunction with the final termination protocol. They underwent the same treatment as the subcutaneous ATs in regards to paraffin embedding and IHC protocols.

Excision biopsies were performed under general anesthesia using three different compounds (Zoletil, Rompun, Ketaminol and Torbugesic) as described previously[1]. The animals were monitored with blood pressure, heart rate and ciliary reflexes to assess anesthetic depth. Any sign of distress was followed by administration of further anesthetic injection.

In the analysis of perivascular AT we took 6 High Power Field (HPF) images of the IHC-stained section with a camera-mounted microscope using the 20x magnification objective, blinded for drug vs. placebo. The images were taken clockwise in reference to the vessel lumen at 2, 4, 6, 8, 10 and 12 o'clock. The images were taken so that no adventitia, other vessels or myocardium were in the HPF and they were maximally 2 HPFs away from the coronary artery wall (adventitia).
Image capture for subcutaneous AT, mesenteric AT and extraperitoneal AT was very similar to that for perivascular AT. 5 HPF images were taken randomly spread out in the tissue sections at 20x magnification. The digital image files were blinded for drug vs. placebo, and for AT compartment. There were no differences in other aspects of analyses.

After capture, images were analyzed with ImageJ v 1.46r (National Health Institute) software using color thresholding to exclude background staining (noise), and only signals with a pixel span of more than 250 coherent pixels were counted as cells. Nearby nucleus was not a criterion for the cell count.

Finally, we visually searched through each image of the CatS stained slides and noted if there were any crown-like-structures present. They were defined as >50% of an adipocyte circumference occupied by CatS positive cells, or >3 CatS positive cells encircling an adipocyte.

Immunohistochemistry

Following deparaffinization and rehydration we performed IHC for CatS and CD163.

<u>CathS</u>: We performed heat-induced antigen retrieval and blocked sections with hydrogen peroxide (DakoCytomation K4011), avidin/biotin and protein (DakoCytomation X0909). We applied a polyclonal goat anti-Cathepsin S antibody (Santa Cruz Biotechnology sc-6505) 2µg/ml for 60 minutes. As control we used goat IgG in identical concentration. We then applied a biotinylated secondary antibody (DakoCytomation multilink E0453) diluted 1:100 for 30 minutes followed by streptavidin-labeled horseradish peroxidase (Vector laboratories SA-5704) for 30 minutes. Staining was detected with 3,3'-diaminobenzidine (DAB), counterstained with haematoxylin after which the slides were dehydrated, cleared and cover slipped.

CD163: We performed proteolytic antigen retrieval (DakoCytomation S3007) for 30 minutes followed by protein blocker (DakoCytomation X0909). We applied a polyclonal rabbit anti-CD163 antibody (Søren K Moestrup, Aarhus University) 0,5μg/ml and incubated the slides overnight at 2-8° C. As control we used rabbit IgG in identical concentration. We then applied a biotinylated secondary antibody (DakoCytomation Multilink E0453) diluted 1:100 for 30 minutes followed by streptavidin-alkaline phosphatase (Vector Laboratories SA-5100) 1:500. Staining was detected with Permanent Red (DakoCytomation K0640) after which the slides were dehydrated, cleared and cover slipped.

Assessment of obesity and adipose tissue distribution

We performed whole-body computed tomography (CT) scanning of all pigs (n=40), 1-8 days before termination on a 50 cm field of view (FOV) Siemens CT scanner or a 70 cm FOV GE Medical Systems PET-CT scanner. CT scan images were exported as DICOM files and evaluated in OsiriX imaging software. A cross-section of the pig was selected at the level of the second lumbar vertebra (L2) or P2 site. The P2 site was defined as the vertebra on the level of the most distal part of the last true rib (Supplementary Fig. 3). As *Val-Laillet et al*[2] have pointed out, the P2 site is selected for 3 reasons. First, it is a site of particular interest in the pig, as this is the most commonly used site for measuring subcutaneous AT by ultrasonography in the pig industry. Second, the widest abdominal girth on minipigs is found between vertebrae T13 and L2, and this holds true for the FBM pig as well. Third, manual delineation of the retroperitoneal space is easy as the kidneys are clearly visible in this slice.

In each pig, we marked a large portion of the subcutaneous AT as a region of interest (ROI) in OsiriX and the mean and SD values in Hounsfield units were noted (Supplementary Fig. 4). We then exported the selected cross-sectional image of the pig to ImageJ for thresholding using the mean +/- 2 SD obtained in the ROI from the same pig. Thereby each pig was used as its own reference for thresholding and identifying AT to avoid implications from using 2 different scanners. The L2 cross-section was cropped into 3 different files. One image file contained the intraperitoneal content, the second contained the retroperitoneal + intraperitoneal area, and the third contained the entire pig (Supplementary Fig. 4).

Thus we measured 3 areas of AT directly: Total AT, intra-abdominal AT, and visceral AT. From these, we calculated another two compartments: subcutaneous AT and extraperitoneal AT.

By outlining the skin on the CT images and measuring the entire area, we obtained the total cross-section area of each pig, and calculated the “lean tissues”. We compared the two groups (drug vs. placebo) with regards to termination body weight, total AT, subcutaneous AT and intraabdominal AT. CT data from one pig, in the placebo group, became corrupt and could not be analyzed.

Statistical analyses

We performed the statistical analyses in GraphPad Prism® 5 for Mac OS X (version 5.0c). Paired or unpaired students t test was used when the data passed the D’Agostino & Pearson omnibus normality test and there was no difference between the variances. When the data did not pass normality or the variances were significantly different, with or without logarithmic transformation, the Mann Whitney test was used for unpaired, and Wilcoxon matched-pairs signed rank test was used for paired data analysis.
When testing for effects of the drug with paired data we calculated the log(W30/W18) of the parameter of interest for each pig, and we tested for a difference between the groups with a t-test or Mann Whitney. When no difference was found, the groups could be combined to find the effects of time/aging by testing if log(W30/W18) was significantly different from zero.

Investigators were blinded for groups when assessing the outcome of all experiments. All p values are two tailed.

References

1. Poulsen CB, Al-Mashhadi AL, Wachenfeldt von K, Bentzon JF, Nielsen LB, Al-Mashhadi RH, Thygesen J, Tolbod L, Larsen JR, Frøkiær J, Tawakol A, Vucic E, Fredrickson J, Baruch A, Frendéus B, Robertson A-KL, Moestrup SK, Drouet L, Falk E (2016) Treatment with a human recombinant monoclonal IgG antibody against oxidized LDL in atherosclerosis-prone pigs reduces cathepsin S in coronary lesions. International Journal of Cardiology 215:506–515. doi: 10.1016/j.ijcard.2016.03.222

2. Val-Laillet D, Blat S, Louveau I, Malbert CH (2010) A computed tomography scan application to evaluate adiposity in a minipig model of human obesity. Br J Nutr 104:1719–1728. doi: 10.1017/S0007114510002667

Supplementary Table 1.

Supplementary statistical results: effects of drug and time/age

IVGTT	Drug effect n	p value	Time/Age effect - W18 vs. W30 log(mean)	n	SD	p value	W30 / W18
HOMA	nD=18, nP=20	0.24	0.092	38	0.210	0.011	1.24
CS_I	nD=17, nP=18	0.54	-0.108	35	0.183	0.001	0.78
AUC - Glucose	nD=17, nP=18	0.84	0.040	35	0.051	<0.0001	1.10
AUC - Insulin	nD=17, nP=18	0.68	0.127	35	0.161	0.0001	1.34

IVGTT	Mean values W(-3)	W30	Time/Age + HFFD - W(-3) vs. W30 log(W30/W(-3))	n	SD	p value	W30 / W(-3)
CS_I	0.066	0.014	-0.65	7	0.24	0.0004	0.22
HOMA-IR	0.97	0.74	-0.13	7	0.24	0.21	0.75
Fasting glucose (mmol/l)	4.9	4.30	-0.05	7	0.05	0.033	0.88
Fasting Insulin (mU/l)	4.3	3.80	-0.07	7	0.23	0.43	0.84
AUC - Glucose (mmol/l * min)	411	950	0.37	7	0.09	<0.0001	2.34
AUC - Insulin (mU/L * min)	591	1449	0.41	7	0.21	0.0023	2.55

Lipids	Time/Age + HFFD - W(-3) vs. W30 log(W30 / W(-3))	n	SD	p value	W30 / W(-3)
Total cholesterol	0.61	38	0.12	<0.0001	4.04
LDL cholesterol	0.63	38	0.14	<0.0001	4.31
HDL cholesterol	0.52	38	0.19	<0.0001	3.29
Triglycerides	0.09	38	0.22	0.013	1.24
LDL-C/HDL-C	0.12	38	0.22	0.002	1.31

Blood pressure	Drug effect n	p value	Time/Age effect - W18 vs. W30 log(mean)	n	SD	p value	W30 / W18
Diastolic	nD=15, nP=17	0.251	-0.025	32	0.130	0.282	0.94
Mean arterial	nD=15, nP=17	0.386	-0.029	32	0.105	0.131	0.94
Heart rate	nD=14, nP=14	0.320	0.027	28	0.097	0.149	1.06
	**n**	**p value**	**median**	**n**	**Wx**	**p value**	**W30 / W18**
Systolic[a]	nD=15, nP=17	0.940	-0.039	32	-258	0.016	0.91

nD = number of drug (MLDL1278A) treated pigs; nP = number of placebo treated pigs; [a]Non-parametric analysis; Wx = sum of signed ranks (Wilcoxon test)

Supplementary Figure 1. Study timeline – interventions and assessments

			W(-3)	W0	W18	W30
Age in weeks:	0	29	35	38	56	68
Diet:	Chow			HFFD		
Intervention:		Castration			Drug trial - (MLDL1278A)	CT-scans
			Fasting lipids (N=38)		Fasting lipids (N=38)	Fasting lipids (N=38)
			IVGTT (N=7)		IVGTT (N=37)	IVGTT (N=38)
					Bloodpressures	Bloodpressures
					Subcutaneous AT biopsies	Subcutaneous AT biopsies
						Necropsy

Supplementary Figure 2. High fat+fructose diet (HFFD)

LabDiet 5081 High Fructose Diet w/ ~22% Fat — 5AW6

DESCRIPTION

Modified LabDiet® Laboratory Mini-Pig Grower Diet 5081 with added Cholesterol, Fructose and Lard.

Storage conditions are particularly critical to TestDiet® products, due to the absence of antioxidants or preservative agents. To provide maximum protection against possible changes during storage, store in a dry, cool location. Storage under refrigeration (2° C) is recommended. Maximum shelf life is six months. (If long term studies are involved, storing the diet at -20° C or colder may prolong shelf life.) Be certain to keep in air tight containers.

*Product Forms Available**	*Catalog #*
3/16" Pellet	1815009

*Other Forms Available On Request

INGREDIENTS

Dehulled Soybean Meal, Lard, Fructose, Wheat Middlings, Ground Oats, High Fructose Corn Syrup - 55, Dehydrated Alfalfa Meal, Dried Beet Pulp, Cholesterol, Calcium Carbonate, Dicalcium Phosphate, Cane Molasses, Salt, Vitamin/Mineral Premix, DL-Methionine, Choline Chloride.

FEEDING DIRECTIONS

Feed ad libitum. Plenty of fresh, clean water should be available at all times.

CAUTION:
Perishable - store upon receipt.
For laboratory animal use only; not for human consumption.

7/29/2011

NUTRITIONAL PROFILE [1]

Protein, %	***17.0***
Arginine, %	1.15
Histidine, %	0.40
Isoleucine, %	0.92
Leucine, %	1.28
Lysine, %	1.01
Methionine, %	0.35
Cystine, %	0.23
Phenylalanine, %	0.81
Tyrosine, %	0.55
Threonine, %	0.65
Tryptophan, %	0.24
Valine, %	0.88
Alanine, %	0.78
Aspartic Acid, %	2.09
Glutamic Acid, %	3.72
Glycine, %	0.80
Proline, %	1.10
Serine, %	0.96
Taurine, %	0.00
Fat (ether extract), %	***22.2***
Fat (acid hydrolysis), %	***23.0***
Cholesterol, ppm	20,201
Linoleic Acid, %	2.55
Linolenic Acid, %	0.17
Arachidonic Acid, %	0.04
Omega-3 Fatty Acids, %	0.17
Total Saturated Fatty Acids, %	9.05
Total Monounsaturated Fatty Acids, %	9.13
Polyunsaturated Fatty Acids, %	2.75
Fiber (max), %	***4.6***
Neutral Detergent Fiber[2], %	11.3
Acid Detergent Fiber[3], %	5.8
Nitrogen-Free Extract (by difference), %	***41.1***
Starch, %	6.29
Glucose, %	2.29
Fructose, %	17.86
Sucrose, %	1.55
Lactose, %	0.00
Total Digestible Nutrients, %	88.3
Energy (kcal/g) [4]	4.33

From:	kcal	%
Protein	0.680	15.7
Fat (ether extract)	2.001	46.3
Carbohydrates	1.645	38.0

Minerals	
**Ash, %**	**5.1**
Calcium, %	0.86
Phosphorus, %	0.51
Phosphorus (available), %	0.28
Potassium, %	0.87
Magnesium, %	0.17
Sulfur, %	0.21
Sodium, %	0.22
Chloride, %	0.36
Fluorine, ppm	14.6
Iron, ppm	231
Zinc, ppm	149
Manganese, ppm	152
Copper, ppm	22
Cobalt, ppm	0.69
Iodine, ppm	1.96
Chromium, ppm	1.21
Selenium, ppm	0.24
Vitamins	
Carotene, ppm	2.0
Vitamin A, IU/g	10
Vitamin D-3 (added), IU/g	5.2
Vitamin E, IU/kg	59
Vitamin K (as menadione), ppm	2.0
Thiamin Hydrochloride, ppm	9
Riboflavin, ppm	9.8
Niacin, ppm	63
Pantothenic Acid, ppm	24
Folic Acid, ppm	9.8
Pyridoxine, ppm	5.55
Biotin, ppm	0.2
Vitamin B-12, mcg/kg	25
Choline Chloride, ppm	1,394
Ascorbic Acid, ppm	0

1. Formulation based on calculated values from the latest ingredient analysis information. Since nutrient composition of natural ingredients varies and some nutrient loss will occur due to manufacturing processes, analysis will differ accordingly. Nutrients expressed as percent of ration on an As-Fed basis except where otherwise indicated. Moisture content is assumed to be 10.0% for the purpose of calculations.
2. NDF = approximately cellulose, hemi-cellulose and lignin.
3. ADF = approximately cellulose and lignin.
4. Energy (kcal/gm) - Sum of decimal fractions of protein, fat and carbohydrate x 4,9,4 kcal/gm respectively.

ISO 9001:2000 CERTIFIED

TestDiet
www.testdiet.com

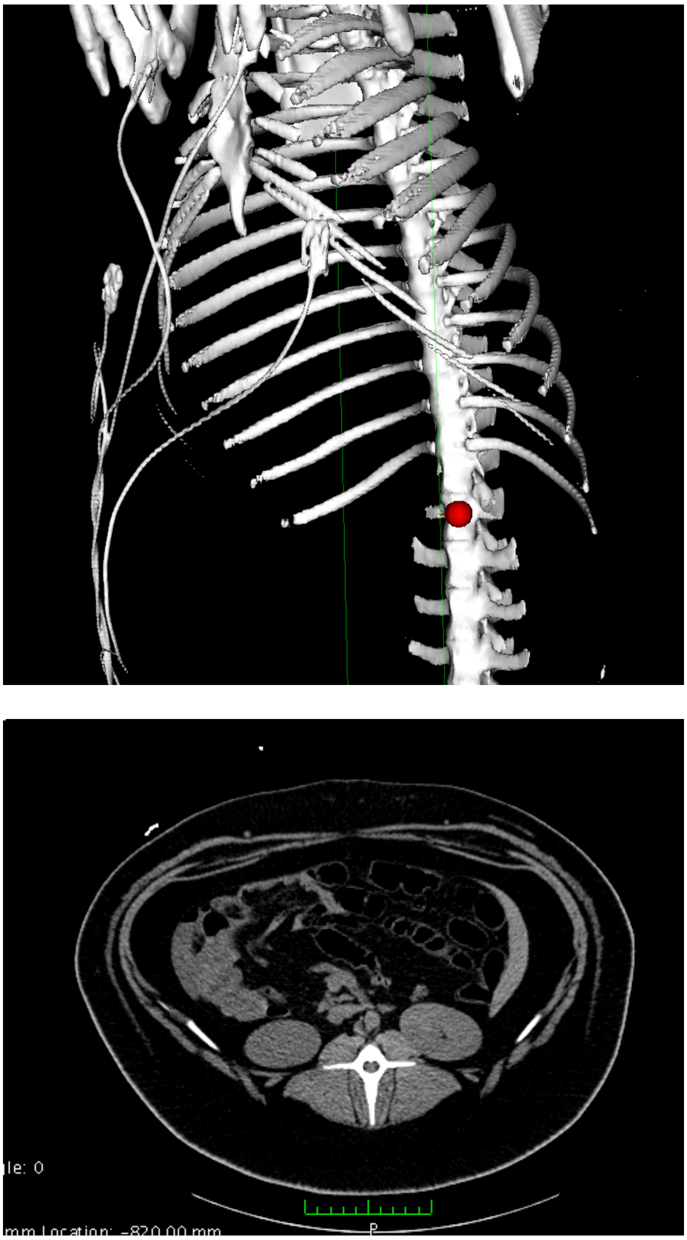

Supplementary Figure 3. Identifying the P2 site by computed tomography imaging.

Top: 3D reconstruction of the FBM-pig spinal column and ribcage. The red dot marks the P2 site corresponding to vertebra L2 right below the last true rib. Bottom: Typical cross-section at the L2 level of an obese FBM pig.

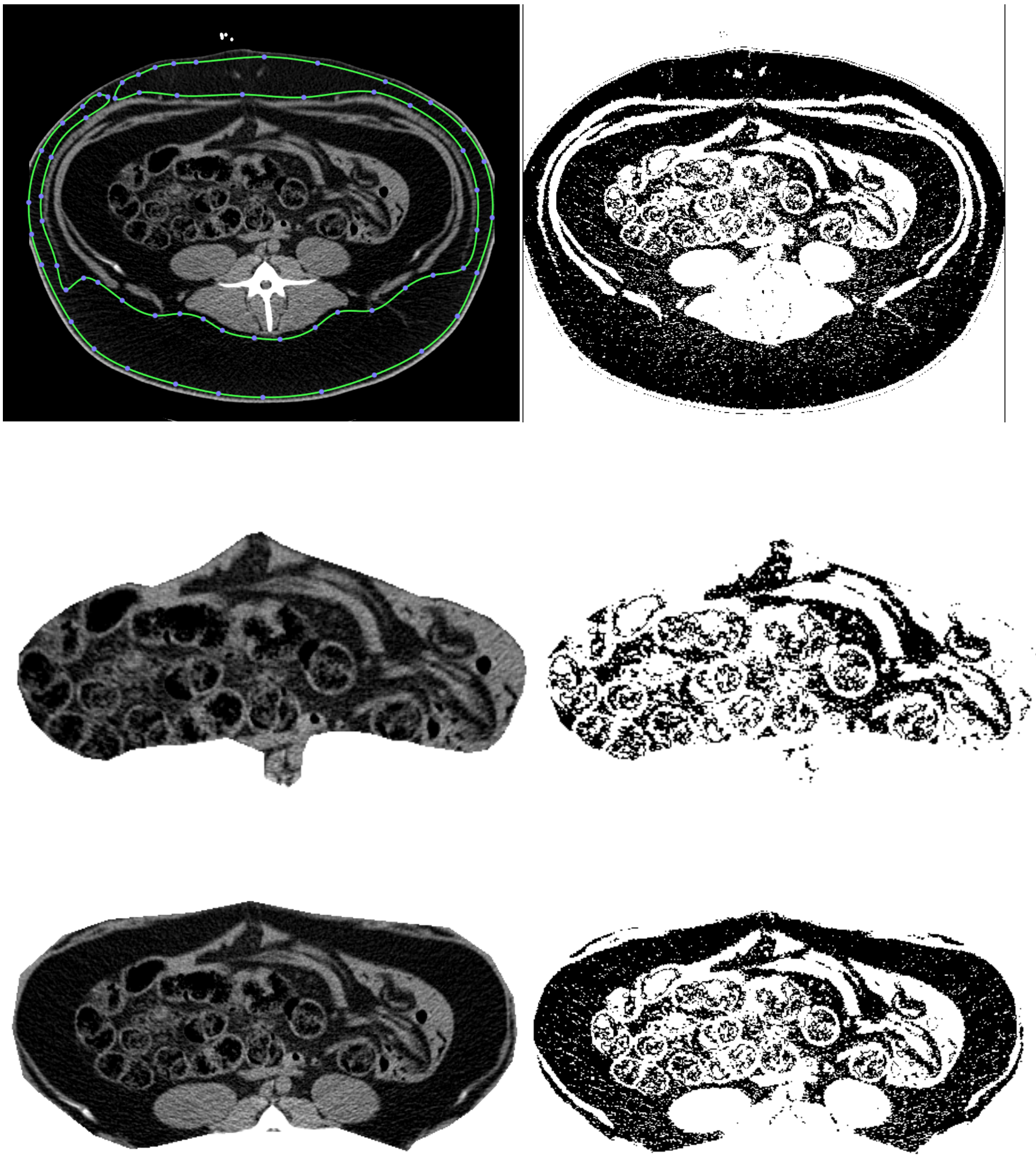

Supplementary Figure 4. Measurement of adipose tissue compartments.
Left column: Cross-sectional computed tomography images at the vertebra L2 level. Right column: Thresholding binary images depicting AT (black). Top row: Total AT. Subcutaneous AT is marked as a ROI in order to obtain the mean and SD values for AT in the pig. Middle row: Intra-peritoneal AT (visceral AT). Bottom row: Intra-abdominal AT.
